# Quantitative wear evaluation of tips based on sharp structures

**DOI:** 10.3762/bjnano.15.22

**Published:** 2024-02-14

**Authors:** Ke Xu, Houwen Leng

**Affiliations:** 1 School of Electrical & Control Engineering, Shenyang Jianzhu University, Shenyang 110168, China

**Keywords:** atomic force microscopy, estimated tip diameter, scanning parameter, tip reconstruction, tip wear

## Abstract

To comprehensively study the influence of atomic force microscopy (AFM) scanning parameters on tip wear, a tip wear assessment method based on sharp structures is proposed. This research explored the wear of AFM tips during tapping mode and examined the effects of scanning parameters on estimated tip diameter and surface roughness. The experiment results show that the non-destructive method for measuring tip morphology is highly repeatable. Additionally, a set of principles for optimizing scanning parameters has been proposed. These principles consider both scanning precision and tip wear. To achieve these results, an AFM probe was used to scan sharp structures, precisely acquiring the tip morphology. Tip wear was minimized by employing lower scanning frequency and free amplitude, and a set point of approximately 0.2, resulting in clear, high-quality AFM images.

## Introduction

AFM is a commonly used multifunctional technology in nanotechnology [1–5]. Compared to optical and electron microscopy, AFM enables three-dimensional (3D) measurements of nanostructures in air and liquid environments [6]. The interaction between the tip and sample influences the measurement results of AFM by convoluting the tip topography with the sample surface topography [7]. A sharper needle tip leads to more accurate measurements [8]. During the scanning process, tip and sample come into mutual contact, causing wear on the tip [9]. Tip wear or damage in practical applications can have severe consequences, including reduced image quality and erroneous data generation [10–11].

Although tip wear is inevitable, optimizing scanning parameters and understanding the wear process can effectively reduce the wear rate [12]. It is essential to accurately determine the tip morphology and analyze the impact of the scanning parameters on tip wear [13]. There are currently two primary methods for obtaining the tip morphology, namely microscopic observation and blind reconstruction based on AFM images. Strahlendorff et al. [12] employed a scanning electron microscope (SEM) to evaluate the shape of the probe before and after scanning to determine tip wear. Orji et al. [14] utilized a transmission electron microscope (TEM) to image a tip and derived its tapered shape from the TEM image. Electron microscopic observation offers the advantages of high precision and resolution, enabling accurate acquisition of morphological information about the tip. However, electron microscope methods have limitations in that they only provide a projected two-dimensional (2D) view of the tip, making in situ measurements impossible. Installation and removal of the AFM tip are time-consuming processes, rendering them unsuitable for widespread use in quantitative tip wear studies. Another approach involves blind reconstruction based on AFM images. Bellotti et al. [15] introduced a uniquely shaped nanoparticle as a tip characterizer. They conducted tip shape analysis using AFM images centered on a single nanoparticle on a flat substrate to investigate the critical size of the reconstructed nanoparticle. Zhang et al. [16] developed a 2 µm lattice sample with uniformity and consistency to reconstruct the AFM tip, thus mitigating the impact of tip effects on measurement results. Onishi et al. [17] proposed a technique to extract the probe shape function from AFM topography images of standard nanoscale spherical particles and rectangular parallelepiped nanostructures of known shapes and to quantitatively estimate the shape of the tip by measuring the known nanostructures in advance. However, tip characterizers with fixed bodies present significant cost and manufacturing challenges. Additionally, uncertainties inherent in simulating the tip characterizer can be transferred to the tip during the characterization process, ultimately affecting the determination of the tip topography.

At the same time, some researchers have studied the impact of scanning parameters on AFM scanning. Xue et al. [18] used SEM to study the state of the probe before and after scanning under different scanning parameters and found that good scanning parameters can be obtained under low scanning speed, large integration gain, and high set point, which significantly reduces tip wear. Su et al. [19] used the estimated tip diameter (ETD) as an indicator to evaluate tip wear and concluded that low set points can significantly reduce tip wear. Also, the probe can reach a predetermined amplitude faster, making faster scanning speeds possible. Huang et al. [20] not only used the ETD as an indicator of tip wear, but also used the surface roughness (*R*_a_) to represent the degree of image deterioration to evaluate the degree of probe wear. It was concluded that a high free amplitude and a set point of 0.5 increase probe wear, while a set point of 0.6 reduces tip wear; the scanning speed does not significantly affect tip wear. The above researchers have studied probe wear under different scanning parameters, but the methods of evaluating wear are not the same, and there are some differences in the results obtained. For example, Xue et al. and Huang et al. concluded that high set points reduce tip wear, while Su et al. concluded that low set points reduce tip wear; Xue et al. believed that scanning speed has a significant impact on tip wear, while Huang et al. believed that scanning speed has little impact on tip wear. Therefore, it is necessary to study the evaluation method of probe wear and analyze the differences in the above research results.

In this paper, a sample with randomly distributed sharp structures is used to characterize the tip morphology. This avoids the influence of uncertainties in the manufacturing process of specific shape characterization samples on tip characterization. Additionally, the use of sharp structures allows for in-situ characterization of the tip, overcoming the disadvantage of needing to disassemble the tip for examination with a scanning electron microscope. Furthermore, this study investigates how scanning parameters, such as free amplitude, scanning frequency, and set point, influence tip wear and image quality. The values of ETD and *R*_a_ determine probe wear in the established probe model. The primary objective of this study is to identify optimal scanning parameters that mitigate tip wear while yielding high-quality AFM images.

## Methods

**Figure 1:**
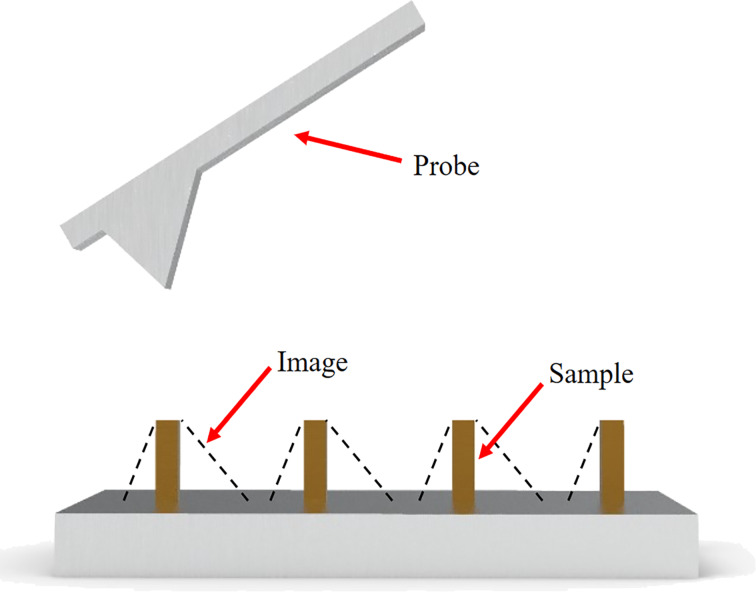
Principle of AFM tip morphology extraction from sharp structures.

The tip morphology can be characterized by its distinct sharp structures, as shown in Figure 1. When the AFM tip scans over a minute columnar protrusion on the substrate, the resulting image contains information about the tip’s structure. By either analyzing the profile of these AFM images or directly observing the finest details of the tip, researchers can glean insights into the tip’s topography and assess its wear.

The image obtained from AFM is influenced by the morphology of the tip, leading to a deviation from the actual sample topography and an “expansion” effect in the scanned image. This phenomenon is commonly known as the broadening effect of the probe tip. In mathematical morphology, the scanning image acquired during the AFM imaging process results from the interaction between sample and tip. The mathematical morphology (Equation 1) can describe the scanning image obtained from AFM. This formula considers both the expansion of the probe and the sample [21],


[1] I=S⊕P.

Here, *P* represents the inversion transformation of the topography curve of the probe centered on the origin, 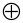 denotes the expansion operator in mathematical morphology, *I* represents the image topography obtained by scanning the sample with AFM, and *S* represents the accurate surface topography of the sample.

An accurate reflection of the tip morphology information is possible when the sample surface *S* exhibits sharp and rapidly changing morphology features. A blind modeling algorithm utilizes the topography information of each pixel and its surrounding pixels during AFM scanning to deduce the local tip topography. Collecting the local tip topography enables a comprehensive calculation of the tip topography.

The tip topography curve can be generated by applying the iterative formula to each point *x* in the acquired AFM scan image, ensuring it satisfies [21],


[2] ∀x∈I,∃d∈P|P⊆I+d−x.

Within the scanned image, the variable *x* represents any point, and *P* refers to the set of displacement changes of the probe tip that we calculate at *x*. By scanning the probe point, we acquire local probe information and use Equation 3 to determine the precise profile of the probe [21],


[3] $$P_{i+1}=\underset{x\in I}{\cap }\left[\left(I-x\right)⊕{P^{\prime }}_{i}\left(x\right)\right]\cap P_{i}.$$

*P_i_*_+1_ is the result of the (*i* + 1)-th iteration operation, 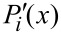 represents the set of displacement changes in the scanning image of the upper bound *P_i_* of the probe calculated at position *x*.

**Figure 2:**
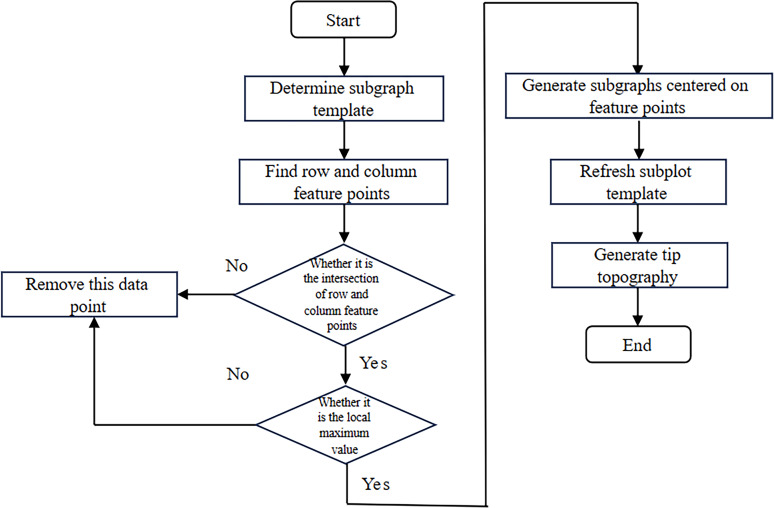
Flowchart for the characterization of tip morphology using sharp structures.

The blind reconstruction algorithm requires the collection of all feature points in the image to determine the probe’s topography accurately. In contrast to the blind reconstruction algorithm, when using sharp structures to characterize the tip topography, the tip morphology is mainly reflected in the sharp feature structures on the sample. Figure 2 illustrates the modeling process.

To enhance accuracy and efficiency of probe modeling, the maximum value in the image is used as the center point of the initial probe template. This ensures that the initial probe template is robust and represents salient features in the image. Then the subgraph template in the image is determined, and the initial template is iterated. Assuming that the image size is *M* × *N* and the initial probe template size is *m* × *n*, the center point of the subgraph *T* must correspond to the local maximum within the subgraph *T*. In other words, the value of the center of *T* should be greater than or equal to the value of its adjacent points in the subgraph, expressed in mathematical terms:


[4]

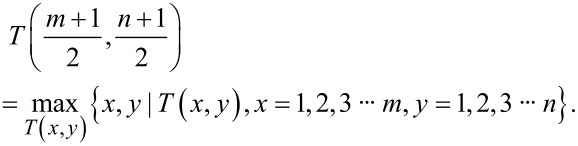



Concurrently, when the central data point of the subgraph corresponds to the local maximum value, it must satisfy the following two conditions. First, it must be the maximum value within its row; second, it must also be the maximum value within its column, as demonstrated in the following formulas,


[5]

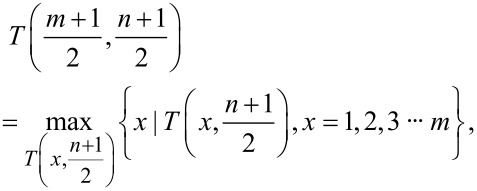




[6]

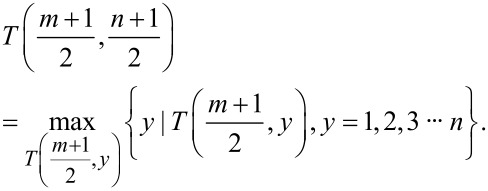



**Figure 3:**
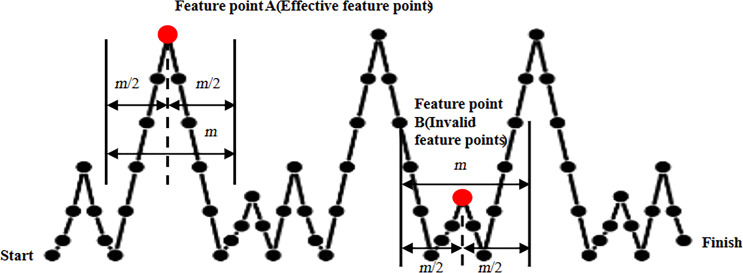
Extraction of scan line feature points.

To determine the subgraph template, it is first necessary to identify the row feature points and column feature points. In Figure 3, the process for identifying feature points in row data is illustrated. Specifically, if the row scan data points within the subgraph template range on both sides of the feature point are smaller than the value of the feature point, then the feature point is considered valid. When a point is deemed a valid feature point, one can directly skip the judgment of the surrounding sub-image template range points, thereby reducing the amount of computation. Conversely, if the surrounding data points exceed the value of the feature point, the point is deemed invalid and should be excluded from subsequent calculations. This process is iterative and requires that the next potential feature point is continually identified. The column feature points can be derived using a similar approach.

By obtaining row and column feature points, the feature point *F* centered on the local maximum of the subgraph is included in the intersection area formed by these peak feature points,


[7] $$F\subseteq \left(R_{i}\cap C_{j}\right)\left(1\leq i\leq M,1\leq j\leq N\right).$$

In this formula, *R_i_* represents the peak feature points for row *i*, while *C_j_* signifies the peak feature points for column *j*. After obtaining the feature point *F*, it needs to be evaluated to determine if it conforms to the local maximum value within the subgraph template. Any points that do not meet this criterion will be excluded. Conversely, those that satisfy the criterion are employed as feature points in the probe model reconstruction to establish the subgraph template. The reconstructed probe template comprises the intersection of all subgraph templates participating in the reconstruction.

## Results and Discussion

### MATLAB implementation of simulating scanned images

In order to simulate the characteristics of a sharp tip, the tapping mode scanning process of an AFM scanning a hard sample in air was simulated in MATLAB. The method of dilation from mathematical morphology was used to simulate the image based on the morphology of the tip and the sample [21]. The convolution effect between the sample and the tip was confirmed, and the potential of using sharp structures to describe the tip structure was examined. Distinct matrix arrays were created for the tip and the sample shapes. To produce an image of a sample matrix using a tip matrix, the tip matrix was positioned perpendicularly above the sample matrix. When the tip apex touched the sample, the difference between the tip and sample matrices was calculated to determine the position of the tip apex. The position of the tip apex was then recorded, and the tip apex was moved horizontally to contact the next pixel of the sample matrix. This process was repeated until all points on the sample matrix had been scanned. The resulting image comprised the recorded height information of the tip apex.

**Figure 4:**
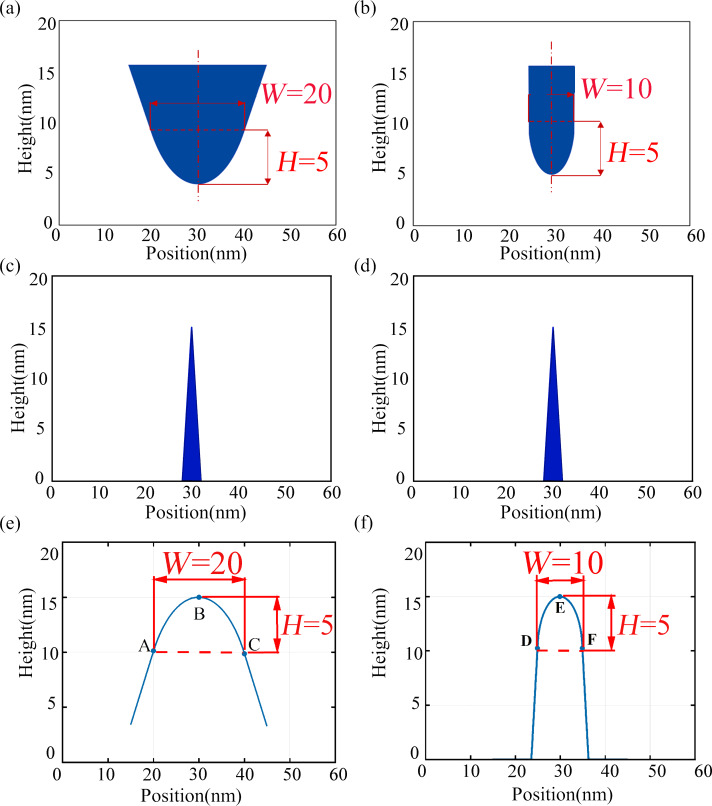
Simulated tip scanning of samples in tapping mode. (a) Blunt tip, (b) sharp tip, (c, d) sharp structure samples, (e) simulated image of a blunt tip scanning a sharp structure, and (f) simulated image of a sharp tip scanning a sharp structure.

Figure 4 illustrates the two tip shapes used in the simulation, a blunt tip with a tip diameter of 20 nm at a distance of 5 nm from the tip apex (Figure 4a), and a sharper tip with a tip diameter of 10 nm also at a distance of 5 nm from the tip apex (Figure 4b). The sample exhibits a prominent sharp structure standing at a height of 15 nm (Figure 4c and Figure 4d). Figure 4a presents the form of the first tip. The top of the tip has a circular cross section, the rearward extension forms a trapezoidal structure, and the two waists are tangent to the top circle. When simulating the scanning of the sample in tapping mode using the blunt tip, the height data graph of the sample forms a continuous arc (Figure 4e). The vertex of the height data plot is labeled as E, and the two data points located 5 nm vertically away from it are labeled as points A and C. The top of the tip consists of a semicircle with a radius of 5 nm, while the part extending backward forms a rectangular structure with two sides tangent to the top circle. Then, we use the rectangular structure’s vertical sides to simulate the ideal sharp probe shape. When scanning a sample using the sharp tip in tapping mode, the resulting graph of sample height data is also a continuous arc. We designate the highest point on the height data plot as E, representing the vertex in the image. Additionally, we label the two data points located 5 nm vertically away from the highest point as points D and F. The distance between point D and point F is 10 nm, which matches the tip diameter of the sharp tip located 5 nm from the apex.

The simulation results show that using a tip to scan a sharp structure can achieve reverse imaging of the tip for both sharp and blunt tips. This inverse imaging of the tip through sharp structures allows for accurate and precise reconstruction of the morphological characteristics of the tips.

### Real data reconstruction results

To analyze the shape of the tip, we use a probe to scan a TipCheck calibration sample and characterize its morphology. The TipCheck sample, provided by BudgetSensors, consists of a silicon chip sample with a wear-resistant thin film coating. The thin film coating exhibits granular and sharp nanostructures, making it ideal for reverse imaging of the AFM probe tip to detect tip wear.

**Figure 5:**
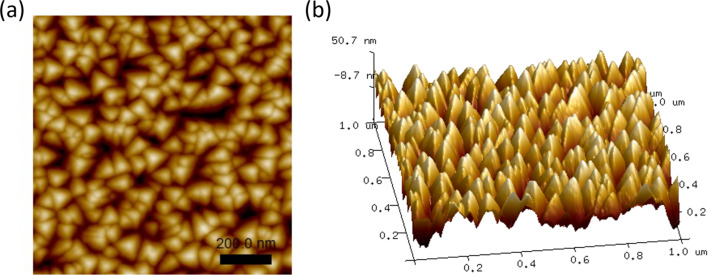
AFM scan images of the TipCheck sample. (a) 2D topography image and (b) 3D topography image.

**Figure 6:**
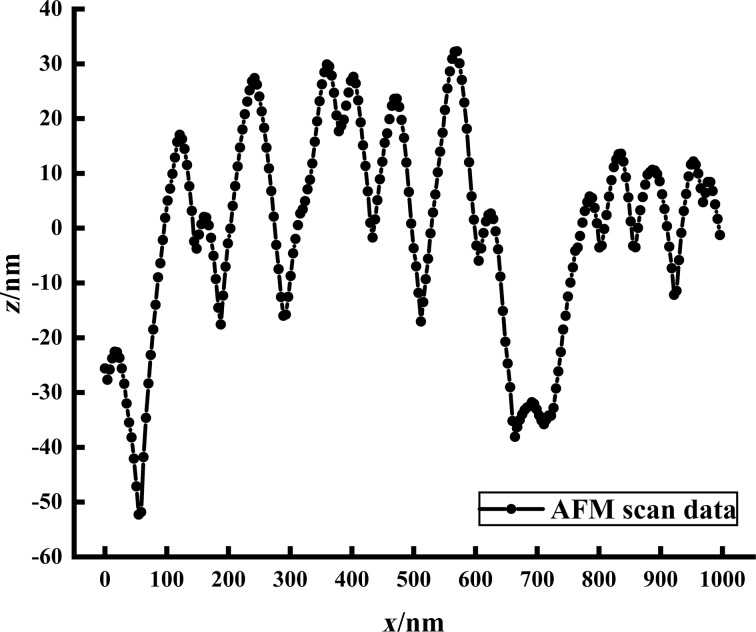
Extraction of scan line feature points.

AFM images were acquired using a Bruker Icon AFM system in tapping mode. The AFM probe used for AFM imaging was a Bruker FESPA-V2 probe. The cantilever was 225 µm long, 35 µm wide, and had a spring constant of 2.8 N/m. The TipCheck sample is extremely sharp, which could cause damage to the tip. Therefore, the needle insertion speed was often reduced, and the initial scanning range was set to 0 µm to prevent damage. The scanning speed should not be too fast, usually 0.5 Hz. As the sample made contact with the surface, the integral gain was gradually increased, and the scanning range was also adjusted accordingly until it reached 1 µm × 1 µm. It was important to set an appropriate scanning range for the AFM image. If the scanning range is too large, it will decrease the accuracy of the image, resulting in a less accurate characterization of the tip. Conversely, if the scanning range is too small, the image might not have enough feature points to characterize the needle tip accurately. Figure 5 shows 2D and 3D images of the TipCheck sample. One of the lines was selected laterally to get the plane view of the 2D TipCheck sample shown in Figure 6. The crest’s location in the plane view indicates potential tips of structures.

**Figure 7:**
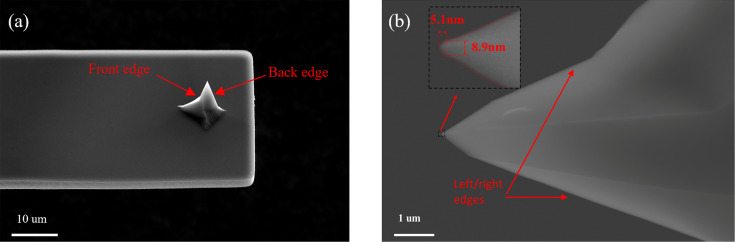
SEM images of AFM tips. (a) Image of tip and cantilever beam and (b) image of the tip; the dotted frame is a partially enlarged view of the tip.

Images of the AFM probes were acquired using a Thermo Fisher Scientific Quattro model scanning electron microscope system. The imaging parameters for the SEM were set to 20 kV electron beam voltage with a working distance of 10–15 mm. Figure 7 presents an SEM image showcasing a probe tip with a rectangular cantilever and a triangular pyramid shape.

**Figure 8:**
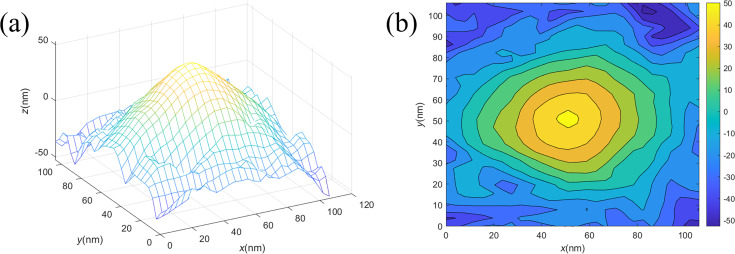
Reconstructed probe model. (a) Reconstructed 3D model of the tip and (b) reconstructed probe contour map.

The actual 3D morphology of the probe obtained using the 3D scanning results of the sample is shown in Figure 8. The analysis of the contour map of the probe model (Figure 8b) concludes that the ETD of the probe is approximately 9.2 nm. This result is similar to the tip diameter result of 8.9 nm in the SEM image shown in Figure 7. The tip results obtained from TipCheck samples are consistent with reality.

**Figure 9:**
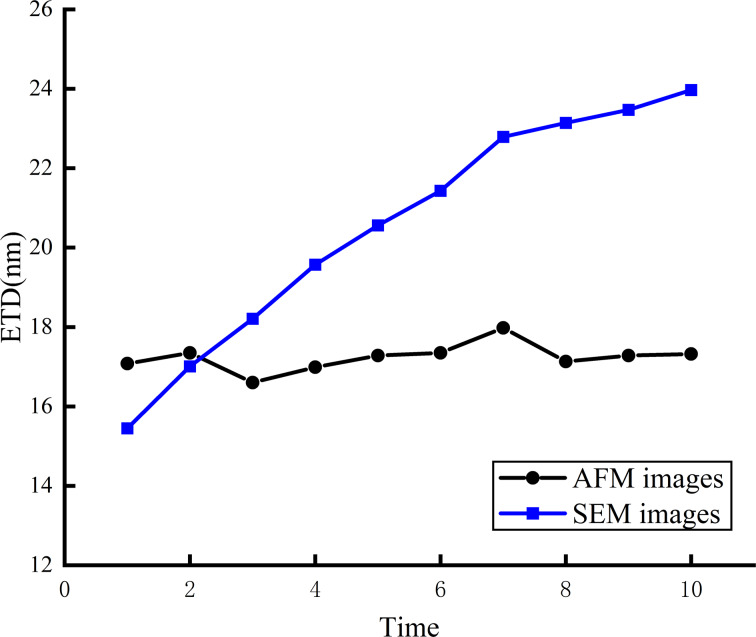
Changes of the ETD determined with AFM and SEM.

SEM and AFM were used to characterize the tip morphology to verify the reliability of the tip morphology determination based on TipCheck samples. The findings in Figure 9 show that as the number of scans increases during SEM observation, the tip diameter fluctuates, and the ETD rises. This observation suggests that a high-intensity electron beam scan may harm the tip and impact the tip morphology measurement. In contrast, there were no substantial changes in the ETD in the morphology characterization results using the TipCheck sample.

The outcomes from TipCheck sample imaging present considerable benefits. This approach significantly reduces the risk of damaging the tip during imaging procedures and simultaneously secures the accuracy of experimental outcomes, producing measurement results that are comparable to those obtained from a scanning electron microscope. Furthermore, this method provides an opportunity for a thorough and precise evaluation of the effects that different scanning parameters have on tip wear. This detailed assessment is instrumental for optimizing scanning conditions, ultimately enhancing the longevity and performance of the AFM tip in various applications.

### Effect of scanning parameters on tip wear

**Figure 10:**
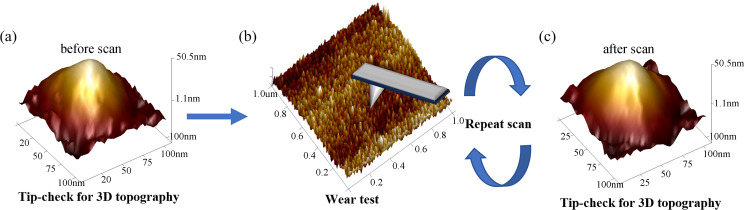
Wear test procedure. (a) 3D reconstruction of the tip topography with the TipCheck sample before scanning, (b) wear test, and (c) 3D reconstruction of the tip topography with the TipCheck sample after scanning.

The wear test procedure comprised three steps, as shown in Figure 10. Each test started with a new AFM tip. First, the TipCheck sample was used to measure the initial tip topography. Then, the wear resistance of the tip was tested by recording the average roughness of each image and conducting regular checks. The 3D topography of the tip was used to determine the ETD with a height of 5 nm, which served as a wear indicator to measure the amount of tip wear. The ETD was a quantitative parameter used to evaluate tip wear during AFM scanning. Comparing the ETD of the tip with its initial diameter helped in determining any alterations caused by wear during the scanning process. An increase in ETD indicated wear and deformation of the tip, while a consistent ETD suggested minimal wear. Additionally, *R*_a_ was utilized to assess changes in image quality resulting from tip wear. As the tip wears, the interaction with the sample surface changes, altering the measured surface roughness. A decrease in *R*_a_ indicates changes of the tip shape due to wear, leading to a decrease in image quality. Conversely, a constant *R*_a_ implies minimal tip wear and a more accurate representation of the sample surface topography. By comparing the initial values of ETD and *R*_a_ with their values after successive scans, researchers can determine the extent of tip wear and its impact on the accuracy of the scanned surface topography. The correlation between changes in ETD and *R*_a_, and their relationship with scan parameters provide a reference for optimizing scan parameters to minimize tip wear while maintaining high-quality AFM imaging.

**Figure 11:**
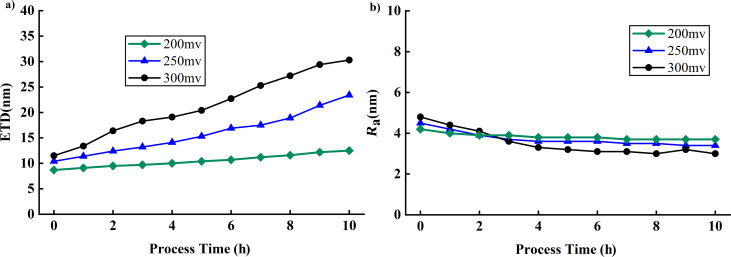
Influence of free amplitude on ETD and *R*_a_. (a) ETD changes at free amplitudes of 200, 250, and 300 mV. (b) *R*_a_ changes at free amplitudes of 200, 250, and 300 mV.

All wear test experiments were conducted using a new needle tip to scan samples of a Si sample coated with gold. The needle tip diameters in each group of experiments were kept similar, avoiding the impact of different initial needle tip diameters on subsequent experiments. In each set of experiments, the effects of tip wear were analyzed by employing various free amplitudes, scanning frequencies, and set points. Additionally, the needle tip diameter was determined every hour. To investigate the impact of free amplitude on wear, each tip was used at a scanning frequency of 1 Hz, with a set point close to 0.5, and a scanning range of 1 µm × 1 µm, ensuring uniformity in the study. Figure 11 illustrates the variation of *R*_a_ and ETD with the free amplitude. At a free amplitude of 200 mV, the ETD increases by 3.8 nm (43.7%), and *R*_a_ decreases by 0.5 nm (11.9%); at a free amplitude of 250 mV, the ETD increases by 13 nm (125%), and *R*_a_ decreases by 1.1 nm (24.4%); at a free amplitude of 300 mV, the ETD increases by 18.8 nm (163.5%), and *R*_a_ decreases by 1.8 nm (37.5%). These results show that the probe wears down as it scans, and the degree of wear increases with higher free amplitude. This increase is due to the increase in tip–sample force when the distance is constant [22–23].

**Figure 12:**
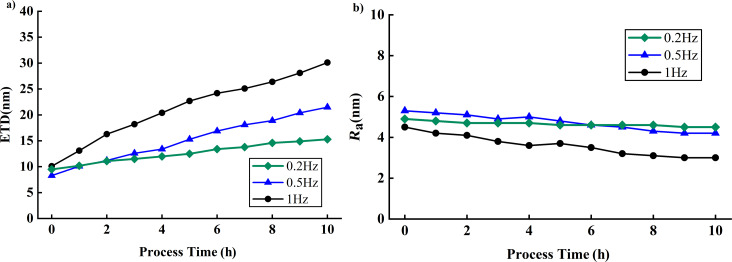
Influence of the scanning frequency on ETD and *R*_a_. (a) ETD changes at line scanning frequencies of 0.2, 0.5, and 1 Hz. (b) *R*_a_ changes at line scanning frequencies of 0.2, 0.5, and 1 Hz.

To investigate the impact of the line scanning frequency on wear, the tip’s free amplitude was maintained at approximately 300 mV, the set point was set to around 0.5, and a scanning range of 1 µm × 1 µm was adopted. Figure 12 illustrates the variations of *R*_a_ and ETD with the scanning frequency. When the line scanning frequency is 0.2 Hz, the ETD increases by 5.8 nm (61.1%) and *R*_a_ decreases by 0.2 nm (4.2%); when the line scanning frequency is 0.5 Hz, the ETD increases by 13.2 nm (155.3%), and *R*_a_ decreases by 1.1 nm (20.8%); when the line scanning frequency is 1 Hz, the ETD increases by 20 nm (198.2%), and *R*_a_ decreases by 1.5 nm (33.3%). When scanning at a frequency of 1 Hz, more significant tip wear was observed compared to 0.2 Hz. This result is consistent with the findings of Xue et al. [18], indicating that faster scanning results in more severe tip wear. However, the experimental results revealed a significant deviation from the findings of Huang et al. [20], which indicated that the scanning frequency had no significant impact on tip wear. In their experiment, Huang et al. set the scanning frequency to 0.2 Hz and 0.4 Hz. At lower scanning frequencies, the tip is better able to track subtle changes on the sample surface, thereby reducing potential damage caused by rapid scanning. Additionally, in Huang et al.’s experiment, the tip had a larger initial diameter at 0.4 Hz. A greater wear resistance of the larger tip, resulting in a relatively lower wear rate at 0.4 Hz, may be assumed. In our experiment, the initial diameters of the tips at different scanning frequencies were very close, yet there was a noticeable increase in scanning frequency from 0.2 Hz to 1 Hz. This suggests that a faster scanning frequency can cause more wear. This increased wear may be attributed to low-cycle fatigue resulting from the higher speeds, which generate more heat and energy dissipation, thereby rendering the tip more susceptible to damage [24].

**Figure 13:**
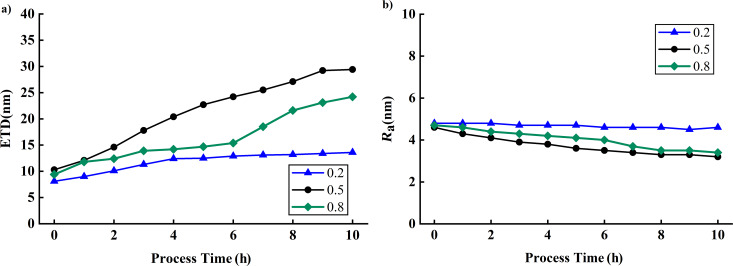
Influence of set point on ETD and *R*_a_. (a) ETD changes at set points of 0.2, 0.5, and 0.8. (b) *R*_a_ changes at set points of 0.2, 0.5, and 0.8.

While varying the set point, a free amplitude of approximately 300 mV was maintained for each tip, a scanning frequency of 1 Hz was set, and a scanning range of 1 µm × 1 µm was used. Xue et al. [18] found that when the set point was low, a significant decrease in the tip’s amplitude could lead to tip breakage during probe engagement. Therefore, in our experiments, we used a higher set point for engaging the probe, ensuring stable contact with the sample surface. Once the tip was stably in contact, we gradually adjusted the set point to avoid damaging the tip during the engagement process. Figure 13 presents the impact of the set point on *R*_a_ and ETD. When the set point is 0.2, the ETD increases by 5.5 nm (67.9%), and *R*_a_ decreases by 0.4 nm (8.2%); when the set point is 0.5, the ETD increases by 19.1 nm (185.4%), and *R*_a_ decreases by 1.4 nm (30.4%); when the set point is 0.8, the ETD increases by 14.8 nm (157.4%), and *R*_a_ decreases by 1.3 nm (27.7%). The results indicate that the needle tip experiences the most severe wear and significant degradation in image quality when the set point is 0.5. Furthermore, the needle tip also suffers from considerable wear when the set point is 0.8. In contrast, minimal impact on image quality is observed at a set point of 0.2, likely due to the reduced impact force resulting from the low set point, thereby reducing tip wear [19]. In the experiment of Huang et al. [20], the initial diameter of the tip at a set point of 0.6 was larger. After two hours of scanning, the tip diameter at a set point of 0.2 approached the initial diameter of the probe at the set point of 0.6. This led to the conclusion that the wear of the tip at a higher set point was less, which might be due to the larger initial diameter of the tip.

Table 1 summarizes the effects of the different scanning parameters on ETD and *R*_a_. When the free amplitude is set to 200 mV, the scanning frequency to 0.2 Hz, and the set point to 0.2, there is a relatively small variation in ETD and *R*_a_. This indicates high stability. These findings underscore the importance of choosing appropriate scanning parameters.

**Table 1:** Impact of scanning parameters on ETD and *R*_a_.

Scanning parameters			ETD change / nm	*R*_a_ change / nm

free amplitude / mV	scanning frequency / Hz	set point		

200	1	0.5	3.8 (43.7%)	0.5 (11.9%)
250	1	0.5	13 (125%)	1.1 (24.4%)
300	1	0.5	18.8 (163.5%)	1.8 (37.5%)
300	0.2	0.5	5.8 (61.6%)	0.2 (4.2%)
300	0.5	0.5	13.2 (155.3%)	1.1 (20.8%)
300	1	0.5	20 (198.2%)	1.5 (33.3%)
300	1	0.2	5.5 (67.9%)	0.4 (8.2%)
300	1	0.5	19.1 (185.4%)	1.4 (30.4%)
300	1	0.8	14.8 (157.4%)	1.3 (27.9%)

**Figure 14:**
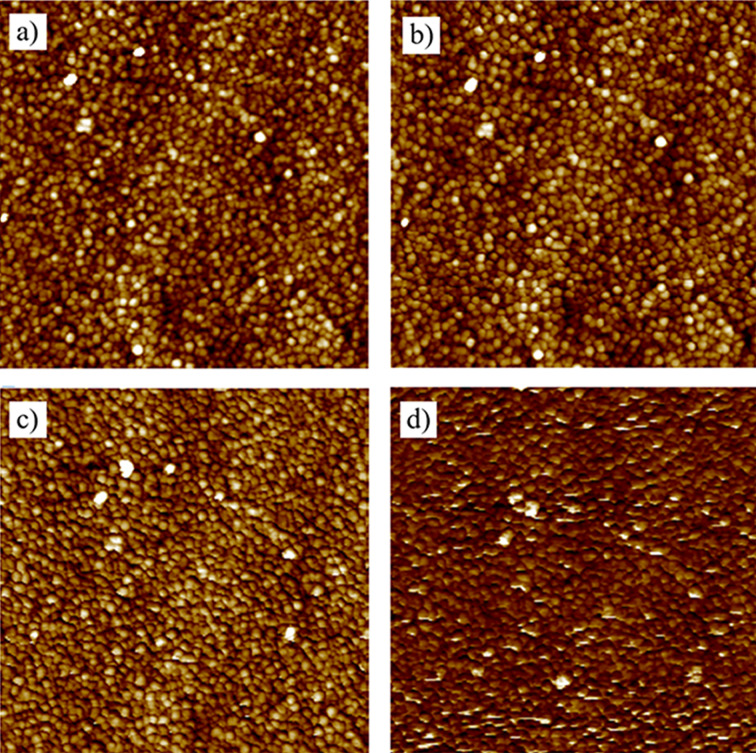
AFM image changes under different scanning parameters. (a) 0 h scan with low-wear settings, (b) 5 h scan with low-wear settings (no noticeable change), (c) 0 h scan with high-wear settings, and (d) 5 h scan with high-wear settings (severe artifacts observed).

Expanding on this, the same sample was scanned using different scanning parameters to assess their impact on image quality. Figure 14 displays images of the identical sample captured with different scanning parameters. After a continuous 5 h scan with parameters set to a free amplitude of 300 mV, a scanning frequency of 1 Hz, and a set point of 0.5, there was a notable decline in image quality. The previously distinguishable granular structure became indistinct, which was attributed to tip wear. However, when the parameters were set to a free amplitude of 200 mV, a scanning frequency of 0.2 Hz, and a set point of 0.2, the image quality remained unchanged after 5 h of continuous scanning. This highlights the importance of determining optimal scanning parameters to limit tip wear and ensure consistently high image quality.

## Conclusion

This paper demonstrates the utility of sharp structures in assessing the topography of AFM tips and delves into the effects of scanning parameters on tip wear. A method employing TipCheck samples has been introduced to determine the shape and structure of AFM tips accurately. Based on its distinct features, the tip morphology can be non-destructively assessed using the AFM imaging mode. This offers a compelling alternative to the conventional SEM detection method. Considering the potential of this technique, it stands on the brink of becoming a standard approach for AFM tip topography detection. The study further examines the impact of various scanning parameters on AFM tip wear, emphasizing tapping mode AFM. ETD and *R*_a_ serve as the evaluation benchmarks. Experimental findings show that free amplitude and scanning frequency significantly affect tip wear and image quality. As amplitude and scanning frequency increase, the wear level also intensifies. The selected set point notably influences ETD and *R*_a_, with pronounced wear detected at a set point close to 0.5. However, lowering the set point proves effective in curtailing tip wear.

## Data Availability

The datasets generated and analyzed during the current study are available from the corresponding author on reasonable request. Further inquiries can be directed to the corresponding author.

## References

[1] Xu K, An Q, Li P (2022). Curr Nanosci.

[2] Cheng C, Wang X, Dong J, Yang F, Ju T, Wang Z (2023). Nanotechnology.

[3] Bai H, Wu S (2021). Microsc Microanal.

[4] Zhou Y, Du J (2022). Prog Biophys Mol Biol.

[5] Wang K, Taylor K G, Ma L (2021). Int J Coal Geol.

[6] Kodera N, Ando T (2022). Curr Opin Struct Biol.

[7] Shen J, Zhang D, Zhang F-H, Gan Y (2017). Appl Surf Sci.

[8] Xu K, Liu Y (2022). Beilstein J Nanotechnol.

[9] Khurshudov A G, Kato K, Koide H (1997). Wear.

[10] Gołek F, Mazur P, Ryszka Z, Zuber S (2014). Appl Surf Sci.

[11] He Y, Zhang L, Cui J, Hu J (2023). Wear.

[12] Strahlendorff T, Dai G, Bergmann D, Tutsch R (2019). Ultramicroscopy.

[13] Chung K-H, Lee Y-H, Kim D-E (2005). Ultramicroscopy.

[14] Orji N G, Dixson R G, Lopez E, Irmer B (2020). J Micro/Nanolithogr, MEMS, MOEMS.

[15] Bellotti R, Picotto G B, Ribotta L (2022). Nanomanuf Metrol.

[16] Zhang X, Zhao L, Han Z, Xu X, Li S, Wu A (2022). Optoelectron Lett.

[17] Onishi K, Fujita D (2011). Anal Sci.

[18] Xue B, Yan Y, Hu Z, Zhao X (2014). Scanning.

[19] Su C, Huang L, Kjoller K, Babcock K (2003). Ultramicroscopy.

[20] Huang Y-P, Lin S-C, Lin V T-Y (2013). Surf Topogr: Metrol Prop.

[21] Villarrubia J S (1994). Surf Sci.

[22] Liu J, Notbohm J K, Carpick R W, Turner K T (2010). ACS Nano.

[23] Vahdat V, Carpick R W (2013). ACS Nano.

[24] Jacobs T D B, Carpick R W (2013). Nat Nanotechnol.

